# An Optimization Method of Production-Distribution in Multi-Value-Chain

**DOI:** 10.3390/s23042242

**Published:** 2023-02-16

**Authors:** Shihao Wang, Jianxiong Zhang, Xuefeng Ding, Dasha Hu, Baojian Wang, Bing Guo, Jun Tang, Ke Du, Chao Tang, Yuming Jiang

**Affiliations:** 1 College of Computer Science, Sichuan University, Chengdu 610065, China; 2 Big Data Analysis and Fusion Application Technology Engineering Laboratory of Sichuan Province, Chengdu 610065, China; 3 Changhong Central Research Institute, Sichuan Changhong Electronic (Group) Co., Ltd., Mianyang 621000, China

**Keywords:** multi-value-chain collaboration, optimal planning, high-dimensional decision space, production-distribution, genetic algorithm

## Abstract

Value chain collaboration management is an effective means for enterprises to reduce costs and increase efficiency to enhance competitiveness. Vertical and horizontal collaboration have received much attention, but the current collaboration model combining the two is weak in terms of task assignment and node collaboration constraints in the whole production-distribution process. Therefore, in the enterprise dynamic alliance, this paper models the MVC (multi-value-chain) collaboration process for the optimization needs of the MVC collaboration network in production-distribution and other aspects. Then a MVC collaboration network optimization model is constructed with the lowest total production-distribution cost as the optimization objective and with the delivery cycle and task quantity as the constraints. For the high-dimensional characteristics of the decision space in the multi-task, multi-production end, multi-distribution end, and multi-level inventory production-distribution scenario, a genetic algorithm is used to solve the MVC collaboration network optimization model and solve the problem of difficult collaboration of MVC collaboration network nodes by adjusting the constraints among genes. In view of the multi-level characteristics of the production-distribution scenario, two chromosome coding methods are proposed: staged coding and integrated coding. Moreover, an algorithm ERGA (enhanced roulette genetic algorithm) is proposed with enhanced elite retention based on a SGA (simple genetic algorithm). The comparative experiment results of SGA, SEGA (strengthen elitist genetic algorithm), ERGA, and the analysis of the population evolution process show that ERGA is superior to SGA and SEGA in terms of time cost and optimization results through the reasonable combination of coding methods and selection operators. Furthermore, ERGA has higher generality and can be adapted to solve MVC collaboration network optimization models in different production-distribution environments.

## 1. Introduction

As global markets become more efficient, competition no longer occurs between individual firms, but it instead occurs across the entire value chain [1], and more and more firms seek to enter the market as an organization in pursuit of greater efficiency in sourcing, production, and distribution. As value chains become larger and more complex in a globalized world, it is important to work efficiently with partners along the chain [2,3,4]. Higher levels of business performance will be achieved by companies that can fully integrate value and supply concurrent streams [5]. In this context, collaboration by two or more companies that create competitive advantage and higher profits than acting alone has been studied by scholars. Ref. [6] divided the collaboration approaches proposed by scholars into vertical collaboration, horizontal collaboration, and lateral collaboration. In [7], it is proposed that lateral collaboration includes the ability of horizontal and vertical collaboration overcoming their limitations, which still requires a lot of research. Besides, the effect of inventory strategy on collaboration was studied in the lateral collaboration model in [7]. Ref. [8] developed a framework based on scenarios designed for collaborative supply chains and then suggested novel models of lateral collaboration to optimize 3BL (triple bottom line) sustainability-related objectives. Ref. [9] modeled the MNE (multi-national enterprise) as a complex adaptive system and investigates the effects of lateral collaboration on performance at both the MNE and subsidiary level. It is found that the existing lateral collaboration models are weak in terms of resource and task allocation and node collaboration constraints in the whole production-distribution process.

In MVC collaboration networks, there is both vertical cooperation on the same chain and horizontal cooperation on different chains, so it is necessary to study the MVC collaboration mechanism and construct an optimization model. Actually, when the value chains operate in collaboration, more collaboration constraints arise and higher requirements are imposed on the collaboration mechanism.

In the MVC collaboration network, there are intersecting nodes between multiple value chains. These nodes represent the enterprises that undertake the tasks of multiple value chains. With the influence of enterprise capacity, warehouse inventory capacity, and other uncertain factors during the operation of the MVC collaboration network, there are many constraints on the intersecting nodes. Such constraints include the time to deliver goods to agents cannot exceed the specified time; production tasks assigned to enterprises in the collaboration network cannot exceed the enterprise capacity; and the number of products stored in the warehouse cannot exceed the upper limit of the storage capacity of the transit warehouse. Therefore, it is necessary to construct a collaboration model to effectively coordinate resources; coordinate the division of labor and the cooperation between enterprises of the value chains; meet the constraints of value chain collaboration; and reduce the internal friction caused by the unreasonable assignment of tasks or resources. 

The production-distribution decision optimization problem of a MVC collaboration network model is a combinatorial planning problem with constraints, while existing research shows that metaheuristic algorithms can solve it effectively. For example, Ref. [10] proposed a service selection model based on collaboration effects and designed an improved GSA algorithm to solve it. Ref. [11] proposed an enhanced multi-objective gray wolf optimizer to solve the manufacturing service optimization combination problem. Therefore, considering the effectiveness and superiority of heuristic algorithms [12,13,14], this study uses genetic algorithms to optimize the production-distribution strategy of MVC collaboration networks. 

In summary, there are few existing studies on resource management and task assignment in MVC collaboration networks. Therefore, the purpose of this paper is to investigate how to adjust the inter-node constraints in value chains after the partners of a MVC have been identified, and to effectively conduct production-distribution planning with the aim of reducing the total production-distribution cost of the collaboration network. To this end, this paper makes the following contributions and innovations:

(1) Taking the definition of collaboration relationship among value nodes in a MVC as the entry point, the collaborative process of the MVC is modeled in the production-distribution scenario of multi-task, multi-production end, multi-distribution end with multi-level inventory in dynamic alliances of manufacturing enterprises. A MVC collaboration network optimization model is constructed with the lowest total production-distribution cost as the optimization objective and with delivery lead time and task quantity as constraints.
(2) A genetic algorithm is used to solve the optimization model, which maps the collaboration constraints of nodes in the value chain to the constraints among genes and solves the conflict problem of MVC collaboration network nodes. In view of the multi-level characteristics of the production-distribution scenario, two chromosome coding methods are proposed in this paper: staged coding, which is applicable to the high requirements for the distribution quantity and time of each stage; and integrated coding, which is applicable to the pursuit of lower cost of the whole production-distribution process.
(3) This paper proposes an ERGA with enhanced elite retention based on SGA. The comparative experiment results and population evolution process show that ERGA outperforms SGA and SEGA in terms of time cost and optimization results through the reasonable combination of coding schemes and selection operators. Besides, ERGA has higher generality and can be adapted to the solution of MVC collaboration network optimization models in different production-distribution environments.

## 2. Related Work

### 2.1. Research Status of Value Chain Collaboration

Along with the internationalization of markets and the enhancement of communication technologies, scholars have proposed a variety of collaboration models. The first is the proposal of a collaboration framework used to build collaboration and partner selection. Ref. [15] developed a virtual e-chain model that presents a supply chain collaboration framework in a virtual environment for classifying the roles of partners, identifying the key capabilities for building each collaboration, and assessing the readiness of partners for collaboration. Ref. [16] developed a theoretical framework on value chains and outlined a value chain process model. To address the multi-cycle and multi-stage problem, ref. [17] proposed an integrated framework to identify effective collaboration mechanisms in a MVC throughout its life cycle. To investigate the collaboration process, scholars have proposed vertical collaboration, horizontal collaboration, and lateral collaboration models. Vertical collaboration can be defined as collaboration when two or more organizations, such as manufacturers, distributors, operators, and retailers, share their responsibilities, resources, and performance information to serve relatively similar end customers. Ref. [18] focused on the process of vertical collaboration between hotels and their food and beverage suppliers, revealing the impact of trust on transaction costs. Ref. [19] developed three different simulation models based on a simple vertical supply chain with one retailer and one operator to investigate the impact of CTM (collaborative transportation management) on total dealer costs and service levels. Horizontal collaboration refers to the collaboration between two or more companies at the same level in a network to facilitate the achievement of common goals, and it can also be referred to as external collaboration. A horizontal collaboration model is proposed using hierarchical analysis to comprehensively assess the degree of collaboration with a single horizontal collaboration program to check the feasibility of meeting customer needs [20]. Lateral collaboration refers to the combination of vertical and horizontal collaboration to obtain increased flexibility and improved resource sharing [21]. Vertical collaboration in supply chains has been investigated intensively in recent years. However, relatively few studies have focused on the horizontal or lateral modes of collaboration [8]. Among them, even less work has been done on lateral collaboration models in terms of resource and task allocation and node collaboration constraints in the whole production-distribution process. However, in the MVC collaboration network for the manufacturing industry, there are often cross nodes between multiple value chains, and the enterprises represented by these nodes undertake multiple tasks division of multiple value chains and have both horizontal and vertical collaboration with other enterprises, so it is necessary to study the MVC collaboration mechanism and construct an optimization model.

### 2.2. Research of Genetic Algorithms

The value chain network design problem in the optimization model is a combinatorial planning problem with multiple constraints, and the metaheuristic algorithm has been proven to be an effective tool to solve the combinatorial planning problem with good performance in many scenarios [22,23,24,25,26,27]. Besides, in recent years, some scholars have combined genetic algorithms into other fields and achieved good results [28,29,30,31].

GAs (genetic algorithms) are a special class of bionic methods that mimic Darwin’s theory of evolution and natural selection, which are well-known population-based metaheuristic algorithms [32,33]. When implementing GAs, the definition of a chromosome, i.e., a string representing a possible solution to a problem, is a key issue that strongly influences the performance of the algorithm in terms of the quality of the optimal solution and the speed of convergence [34]. Encoding strategies are widely used today, such as real-valued encoding, Prufer encoding, and priority-based encoding [35]. The most important operator in genetic operators is the selection operator, which is the key factor affecting the performance of GAs [36]. For common selection operators, such as the roulette wheel operator and the tournament selection operator [37,38], they cannot guarantee that the best-performing individuals can be left in each evolution, while the advantage of the elite retention strategy is that the best individuals of a certain generation cannot be destroyed by genetic operations during the search process, which can guarantee the convergence of genetic algorithms [39]. Therefore, to improve the convergence of the algorithm, the elite retention strategy has been combined with the selection strategy [40]. Genetic operators, including crossover and mutation, are then applied with some probability to the chromosomes to create new and potentially better solutions. The main purpose of crossover operations is to enable the algorithm to explore the search space more efficiently and to produce better individuals in the next generation [41]. However, for some specific problems, the crossover may produce infeasible solutions, so the crossover operator needs to be selected for specific problems, such as the cryptography problem [42]. In conclusion, genetic algorithms need improvement in specific application scenarios, so this paper investigates the combination of coding and operators in a MVC collaboration optimization model.

## 3. Optimization Model of MVC Collaboration Network

### 3.1. Model Mathematical Symbols

The meanings of mathematical symbols used in the paper are shown in the following Table 1.

### 3.2. Value Node Collaboration Definition

The essence of MVC collaboration is the collaboration among value nodes, and the collaboration strategy is the central issue of value chain collaboration, including the collaboration of the division of labor of each link in the value chain, such as production collaboration strategy, sales collaboration strategy, logistics collaboration strategy, and so on. To better construct a model of the MVC collaboration network, the following value-node collaborative relationship definitions are given.

**Definition** **1.** *Collaboration between multiple value chains. Define the value chain set $VC=\left\{VC_{1},VC_{2},\ldots ,VC_{n}\right\}$* *as n* *value chains that operate collaboratively. Each value chain is composed of multiple value nodes, the value node can be an enterprise, a production line, a workshop, and other units that can complete value-added activities. If $VC_{i}=\left\{n_{i}^{1},n_{i}^{2},\ldots ,n_{i}^{m}\right\}$* *, $VC_{i}\in VC$* *denotes that the i* *-th collaborative value chain consists of m* *nodes. $n_{i}^{1}$* *and $n_{i}^{m}$* *respectively represent the starting point and ending point of the value chain $VC_{i}$* *. If $VC_{i}\cap VC_{j}=\left\{n_{i}^{1},n_{i}^{2},\ldots ,n_{j}^{1},n_{j}^{2}\right\}$* *,* *$VC_{i},VC_{j}\in VC$* *, then value node $n_{i}^{1},n_{i}^{2},\ldots ,n_{j}^{1},n_{j}^{2}$* *simultaneously undertake the labor of the value chain $VC_{i}$* *and $VC_{j}$* *,* *which are the intersection nodes of the value chains.*

**Definition** **2.** *Collaboration within the value chain. There are many mixed relationships in the product production process. The value chain composition includes parallel, selection, loop, and other mixed link structures, so there are also collaboration relationships on the same value chain. $CRv(n_{l}^{i},n_{l}^{j})=1$* *means the i* *-th and the j* *-th value node of the l* *-th value chain have a collaboration relationship. Inversely, $CRv(n_{l}^{i},\;n_{l}^{j})=0$* *means they do not have collaboration relationship.*

**Definition** **3.** *Collaboration relationship between nodes of multiple value chains. $n_{a}^{i}\;$* *and $\;n_{b}^{j}$* *are value nodes of value chain $VC_{a}$* *and $VC_{b}$* *, respectively, and the corresponding types of value-added services are $s_{a}$* *and $s_{b}$* *. When $s_{a}=s_{b}$* *, the node $n_{a}^{i}\;$* *and the node $n_{b}^{j}$* *belong to the same value-added service type, i.e., their capabilities are similar, so they have the basis of collaboration. That is, with certain conditions, they can cooperate. The synthetic relation can be described as $CRv(n_{a}^{i},\;n_{b}^{j})=1$* *. When there is no collaboration relationship between nodes, $CRv(n_{a}^{i},\;n_{b}^{j})=0$* *. Extend this relation to the whole network, $CRv\left\{n_{a}^{i},\;n_{b}^{j},\ldots ,n_{c}^{k}\right\}=1$* *means the node $n_{a}^{i},\;n_{b}^{j},\;\ldots ,\;n_{c}^{k}$* *have collaboration relationship among them. $n_{a}^{i},\;n_{b}^{j},\;\ldots ,\;n_{c}^{k}$* *are different value nodes in different value chains.*

The essence of MVC collaboration lies in the collaboration between value chain nodes to achieve win-win results. Through the above definition, the collaboration relationship between nodes on the same chain and the collaboration relationship between nodes on different chains are described.

### 3.3. Description of Collaboration Network Scenario

**Figure 1:**
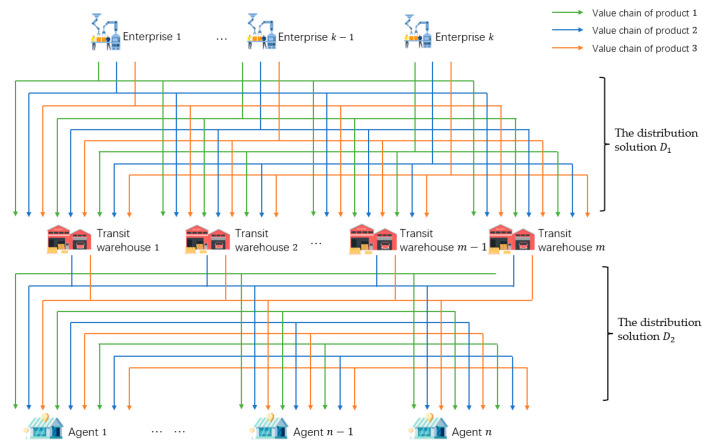
The business scenario of a MVC collaboration network.

The MVC collaboration network as shown in Figure 1 consists of enterprises, transit warehouses, logistics, and agents. There are three kinds of products in Figure 1, so there are three value chains ($VC_{1}$, $VC_{2}$, and $VC_{3}$) operating collaboratively.

Taking $VC_{1}$ and $VC_{2}$ as an example, the process of obtaining the solution can be divided into three steps. Firstly, the agents order the products, then the task-issuing platform summarizes the order information. Next, according to the order information, the platform chooses $n_{1}^{i},\;n_{1}^{j},\;\ldots ,\;n_{2}^{k}$ from the value nodes with collaborative basis, i.e., $CRv\left\{n_{1}^{i},\;n_{1}^{j},\;\ldots ,\;n_{2}^{k}\right\}=1$, on $L_{1}$ and $L_{2}$ to complete the orders. Then, based on the information of each node, the solution with the minimum total cost of the collaboration network is formulated. The solution can be divided into two parts, the first part is the production solution, and task issuing platform generates task sets for enterprises according to production capacity. The other part is the distribution solution. Enterprises will produce the specified products within a certain period. If each enterprise directly transfers the products to the nearest transit warehouse, it may not be able to store the products due to the limited storage capacity of the warehouse, which will lead to a conflict between manufacturing nodes and transit nodes. Therefore, it is necessary to find a product distribution solution on the premise of not exceeding the upper limit of each warehouse capacity and meeting the order quantity and time required by agents. The distribution solution includes two stages, that is, the first stage of distribution solution ($D_{1}$) from the enterprises to the transit warehouses, and the second stage of distribution solution ($D_{2}$) from the transit warehouses to the agents.

The overall distribution route of the product is shown in Figure 1. The products produced by enterprises are distributed to the corresponding transit warehouses for storage according to the distribution solution $D_{1}$. The products stored in the transit warehouses are then distributed to the designated agents according to the distribution solution $D_{2}$ and complete a transaction.

### 3.4. Assumptions and Descriptions

The MVC collaboration network model is constructed based on the following assumptions:

(1) The types and quantities of products produced by the enterprise alliance are equal to the types and quantities in the orders of the agents.
(2) The total time of production and distribution shall not exceed the maximum lead time ($T_{a_{i}}$) of the order.
(3) The tasks assigned to the enterprise shall not exceed the upper limit of the production capacity of them, and the number of goods distributed by the enterprises to the transit warehouses shall not exceed the upper limit of the storage capacity of the transit warehouses.
(4) The distribution cost and time of a single item from the enterprise to the transit warehouse are known and fixed; the delivery cost and delivery time of a single item from the transit warehouse to the agent are also known and fixed.
(5) There is no secondary processing of manufactured products. The route from the warehouse of enterprises to the transit warehouse and then to the warehouse of agents only has three links: the warehouse of enterprises, the transit warehouse, and the warehouse of agents. Each link only goes through once and there is no loop.
(6) The selected enterprises can meet the product production requirements put forward by agents.
(7) Each value chain of the collaboration network is represented by the value increment process of each product, and there are inter-value-chain and intra-value-chain collaborations in the network.
(8) To meet the customized requirements of agents, the manufacturing of products often involves specific parameter requirements, so the production is not in advance.

### 3.5. Decision Variables

In the MVC collaboration operation, there is a decision space composed of the production solution and the distribution solution, which has a decisive influence on the production-distribution cost. There are $P_{tol}$ types of products, $E_{tol}$ manufacturers, and $C_{tol}$ agents in the MVC collaboration network model. The production solution of enterprise i is represented as shown in Equation (1).

(1) $$mq_{e_{i}}=\left(Q_{e_{i}}^{1},Q_{e_{i}}^{2},\ldots ,Q_{e_{i}}^{p}\right)$$

The overall production solution of the MVC collaboration network is represented as shown in Equation (2).

(2) $$MQ=\left[\begin{array}{ccc} Q_{e_{1}}^{1} & \cdots & Q_{e_{1}}^{p}\\ \vdots & \ddots & \vdots \\ Q_{e_{m}}^{1} & \cdots & Q_{e_{m}}^{p} \end{array}\right]$$

The distribution solution $D_{1}^{p}$ of product p in the first stage of the MVC collaboration network is shown in Equation (3).

(3) $$D_{1}^{p}=\left[\begin{array}{ccc} d_{e_{1}}^{t_{1}} & \cdots & d_{e_{1}}^{t_{j}}\\ \vdots & \ddots & \vdots \\ d_{e_{i}}^{t_{1}} & \cdots & d_{e_{i}}^{t_{j}} \end{array}\right]$$

The distribution solution $D_{2}^{p}$ of product p in the second stage of the MVC collaboration network is shown in Equation (4).

(4) $$D_{2}^{p}=\left[\begin{array}{ccc} d_{t_{1}}^{c_{1}} & \cdots & d_{t_{1}}^{c_{j}}\\ \vdots & \ddots & \vdots \\ d_{t_{i}}^{c_{1}} & \cdots & d_{t_{i}}^{c_{j}} \end{array}\right]$$

### 3.6. Optimization Objective Function

The MVC collaboration network is optimized mainly from the perspective of cost based on the actual production-distribution situation. Therefore, the minimum total cost TC is select as the optimization objective function, which is composed of the total production cost and total distribution cost.

The total cost in the collaboration model should include the cost of PSs (production sites) and DCs (distribution centers), etc., and the cost of PSs includes the operating and manufacturing cost [8]. But this paper main focus of study is the optimization of production-distribution strategy, so the total production cost (TPC) is divided into total variable cost and total fixed cost, and the formula is shown in Equation (5). The total fixed cost represents the sum of the fixed inputs produced during a period, such as a series of expenses incurred in the operation of a production plant, which does not vary with the number of products produced. The total variable cost will increase with the increased number of products produced by the enterprises. The expression is shown in Equation (6).

(5) TPC=TVC+TFC

(6) $$TVC=F_{1}\left(Q\right)$$

Total distribution cost (TTC) consists of two parts: one is the distribution cost generated by shipping from the enterprises to the transit warehouses, and the other is the distribution cost generated by shipping from the transit warehouses to the agents. To simplify distribution cost calculation, batch pricing is adopted, and distribution cost is positively correlated with distribution route length and product quantity [35], as shown in Equation (7).

(7) $$TC=F_{2}\left(Q,L\right)$$

The matrix of path length L is shown in Equation (8).

(8) $$L=\left[\begin{array}{ccc} l_{1}^{1} & \cdots & l_{1}^{j}\\ \vdots & \ddots & \vdots \\ l_{i}^{1} & \cdots & l_{i}^{j} \end{array}\right]$$

In the MVC collaboration network model, the quantity and variety of manufactured products are determined by the orders of agents. Therefore, when the quantity of products is fixed, the distribution route solution is the decisive factor affecting the distribution cost and the production solution is the decisive factor affecting the production cost. Therefore, the optimization objective function of total production cost and total distribution cost can be obtained as Equation (9).

(9) $$\left\{\begin{array}{c} \begin{array}{c} minTC=min\left(TMC+TTC\right)\\ TPC=\sum_{p\in P}\;\sum_{e\in E}F_{1}\left(Q_{e}^{p}\right)+TFC\\ TTC=\sum_{k=1}^{P_{tol}}\sum_{j=1}^{T_{tol}}\sum_{i=1}^{E_{tol}}\;F_{2}\left(D_{1}^{p_{k}}{}_{ij},\;l_{e_{i}}^{t_{j}}\right)+\sum_{k=1}^{P_{tol}}\sum_{j=1}^{C_{tol}}\sum_{i=1}^{T_{tol}}F_{2}(D_{2}^{p_{k}}{}_{ij},l_{t_{i}}^{c_{j}}) \end{array} \end{array}\right.$$

### 3.7. Constraint Condition

In the operation of the MVC collaboration network, there are many constraints: the capacity of enterprises; the inventory capacity of transit warehouses; and uncertain factors. According to our assumptions in Section 3.4, the important constraints set in the model are as follows:

1. The enterprises in the MVC collaboration network cooperate to complete all the agents’ orders, and the variety and quantity of the products must meet the agents’ order requirements.
  (10) $$\forall p\in P,\sum_{i=1}^{M_{tol}}Q_{e_{i}}^{p}=\sum_{j=1}^{C_{tol}}Q_{c_{j}}^{p}$$
2. In Equation (10): $\overset{M_{tol}}{\underset{i=1}{\sum }}Q_{e_{i}}^{p}$ represents the sum of the quantity of product p produced by enterprises; $\overset{C_{tol}}{\underset{j=1}{\sum }}Q_{c_{j}}^{p}$ represents the agents’ total need of the quantity of product p.
3. The agent’s order has a maximum lead time ($T_{a_{i}}$), that is, the time taken from production to delivery to the agents has to less than $T_{a_{i}}$. Enterprises are limited by site scale, human resources, production equipment, and other resources, and each enterprise has a limit of daily production capacity. Therefore, the production tasks assigned to enterprises in the collaboration network cannot exceed their production capacity. Production time is positively correlated with daily production capacity, and the distribution time is positively correlated with the distribution route length. Since agent orders cannot be split, products required by agent $a_{i}$ will be uniformly sent out after each enterprise finishes production and delivery to the designated transit warehouse, and the following time constraint (Equation (11)) will be imposed on each agent’s order:
  (11) $$\left(T_{e}^{p_{i}}+T_{tw}^{e}+T_{a_{i}}^{tw}\right)\leq T_{a_{i}}$$
4. Limited by the scale and supporting resources, the storage capacity of the transit warehouses is limited, and the number of stored products cannot exceed the upper limit of the storage capacity of the transit warehouses Equation (12).
  (12) $$\forall tw\in TW,\sum_{i=1}^{P_{tol}}Q_{tw}^{p_{i}}\leq MaxQ_{tw}$$
5. The quantity of products sent by the transit warehouse to the agent, i.e., the export of the transit warehouse, depends on the number of products sent by the enterprise to the transit warehouse Equation (13), i.e., the import of the transit warehouse.
  (13) $$\sum_{i=1}^{E_{tol}}d_{e_{i}}^{tw_{j}}=\sum_{k=1}^{A_{tol}}d_{tw_{j}}^{a_{k}}$$

Therefore, the constraints existing in the MVC collaboration network are shown in Equation (14).

(14) $$S.T.\;\left\{\begin{array}{c} \begin{array}{c} \sum_{i=1}^{E_{tol}}Q_{e_{i}}^{p_{i}}=\sum_{j=1}^{A_{tol}}Q_{a_{j}}^{p_{i}}\\ \left(T_{e}^{p_{i}}+T_{tw}^{e}+T_{a_{i}}^{tw}\right)\leq T_{a_{i}}\\ \forall tw\in TW,\sum_{i=1}^{P_{tol}}Q_{tw}^{p_{i}}\leq MaxQ_{tw}\\ \sum_{i=1}^{E_{tol}}d_{e_{i}}^{tw_{j}}=\sum_{k=1}^{A_{tol}}d_{tw_{j}}^{a_{k}} \end{array} \end{array}\right.$$

The MVC collaboration network scenario is described in this chapter. Based on the analysis of the collaboration relationship and the production-distribution business scenario of MVC in enterprise dynamic alliance, the optimization model of the MVC collaboration network is constructed.

## 4. Algorithm for Optimizing the MVC Optimization Model

The above model is a single objective optimization problem with constraints. In the model constructed, the scale of solution space will increase exponentially with the increase of decision variables caused by the increased number of product types, enterprises, warehouses, and agents. It is difficult to solve the problem with conventional solution methods, so the evolutionary algorithm is adopted to solve it. Evolutionary algorithms (EAs) have a long history of successfully solving COPs (constrained optimization problems) [43]. One of the EAs, the GA, is a probability-based random search algorithm [44], which can search for the optimal solution or sub-optimal solution in the solution space, and is an effective method to solve the optimization problem with a high-dimensional solution space [45].

### 4.1. Algorithm Flow

**Figure 2:**
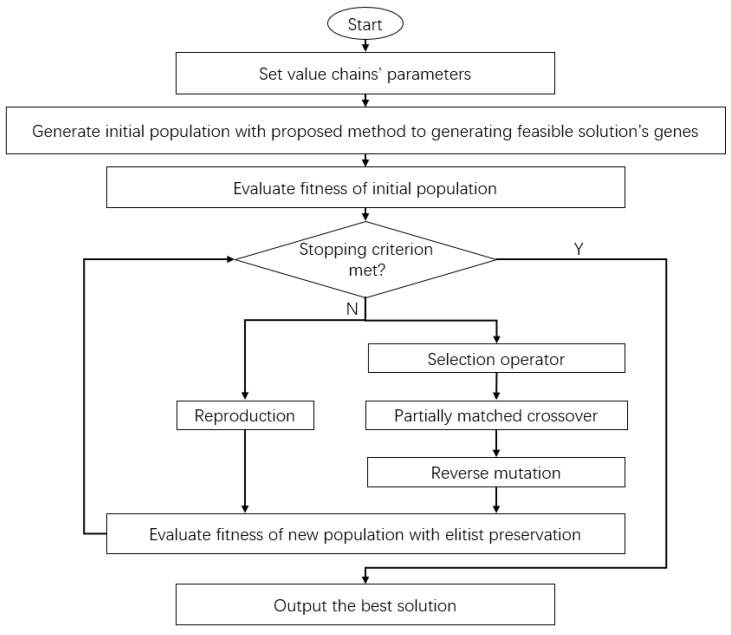
Process of the genetic algorithm.

The process of the genetic algorithm adopted is shown in Figure 2. After setting the node collaboration relationship and constraint conditions of the model, the production-distribution solution that minimizes the total cost of the MVC collaboration network model can be obtained by the algorithm. To select the appropriate encoding scheme and selection operator, the encoding scheme and selection operator will be determined after comparative experiments.

### 4.2. Chromosome Encoding

Chromosome encoding is designed to match genes with solutions. There are several chromosome encoding methods, such as binary encoding and real-value encoding. To improve the encoding feasibility and expansibility [33], real-value encoding is adopted.

Considering the characteristics of the product solution and distribution solution, the high-dimensional decision variable matrix composed of production decision variables and distribution decision variables shown in Equations (2)–(4) is converted into a one-dimensional matrix, which is represented by integer encoded chromosomes. Since multiple products on multiple value chains are involved, and the logistics part of this scenario is a two-stage distribution problem, before the encoding of the logistics solution of the two stages, the following two issues need to be taken in account: (1) how to meet the constraints of the two stages; (2) whether the encoding method used has high solving efficiency and high-quality solution. According to these, two encoding methods are proposed: staged encoding and integrated encoding.

**Figure 3:**
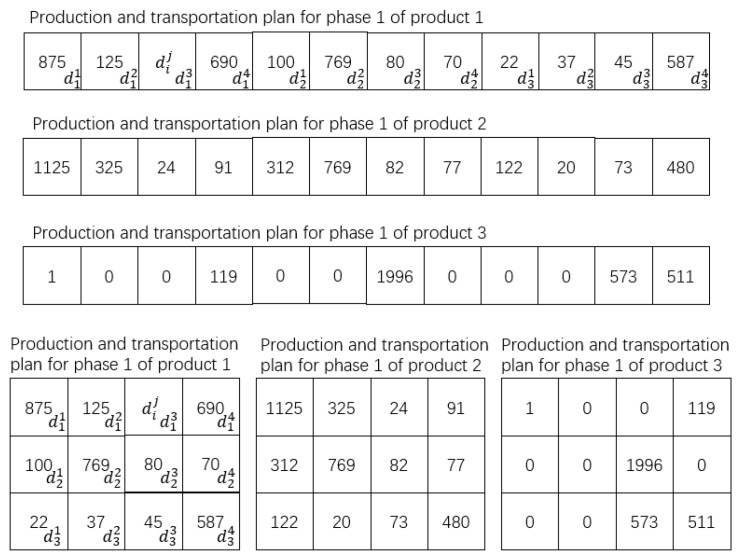
Encoding of solutions in the first stage.

Method (1): staged encoding, that is, the genetic algorithm is used to obtain the optimal distribution solution of the first stage firstly, and the solution of the first stage is used as conditions to obtain the solution of the second stage. Three products, three enterprises, four transit warehouses, and three agents were taken as conditions to build an MVC collaboration network model. The possible values of each group of decision variables MQ and $D_{1}$ of the first stage were represented by one-dimensional decimal chromosomes, as shown in Figure 3. 

In the MVC collaboration network model, there are 3 enterprises and 4 transit warehouses in the distribution process of the first stage. The genes in the chromosome represent the number of products delivered from the enterprise to the transit warehouse, the subscript represents the serial number of the corresponding enterprise, and the superscript corresponds to the serial number of the transit warehouse. 

In Figure 3: $d_{i}^{j}$ is part of the distribution solution of the i-th enterprise for illusion, in other words, it is the number of products distributed from the i-th enterprise to the j-th transit warehouse. $\overset{TW_{tol}}{\underset{j=1}{\sum }}d_{i}^{j}$ is the total number of products produced by enterprise i, i.e., the production solution of enterprise i.

**Figure 4:**
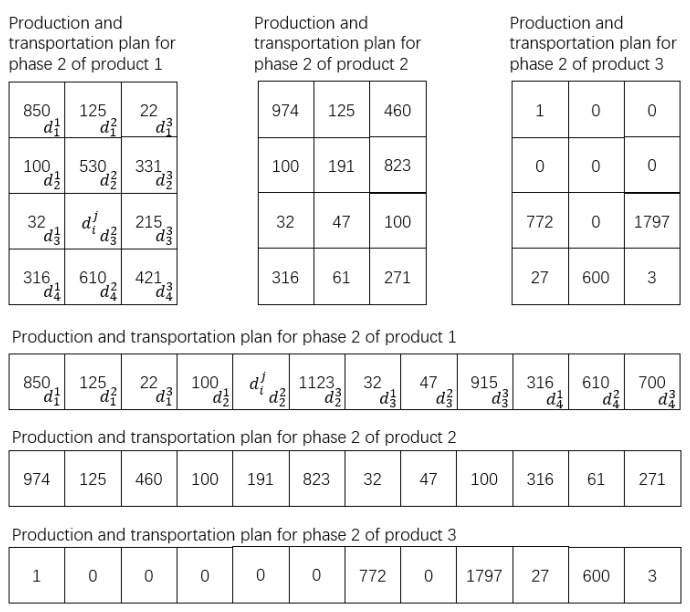
Encoding of solutions in the second stage.

The second stage decision variable consists only of $D_{2}$, and the possible values for each group of $D_{2}$ are represented by the one-dimensional decimal chromosomes, as shown in Figure 4.

In the MVC collaboration network model, there are 4 transit warehouses and 3 agents in the second stage of the distribution process. The genes in the chromosome represent the number of products transferred from the warehouses to the agents, the subscript represents the serial number of the corresponding transit warehouse, and the superscript corresponds to the serial number of the agent manufacturer.

In Figure 4: $d_{i}^{j}$ is the distribution solution of the i-th transit warehouse, in other words, it is the total number of products distributed from the i-th transit warehouse to the j-th agent. $\overset{TW_{tol}}{\underset{j=1}{\sum }}d_{i}^{j}$ is the total number of products distributed by transit warehouse i.$\;\overset{A_{tol}}{\underset{i=1}{\sum }}d_{i}^{j}$ is the total number of products received by agent j.

**Figure 5:**
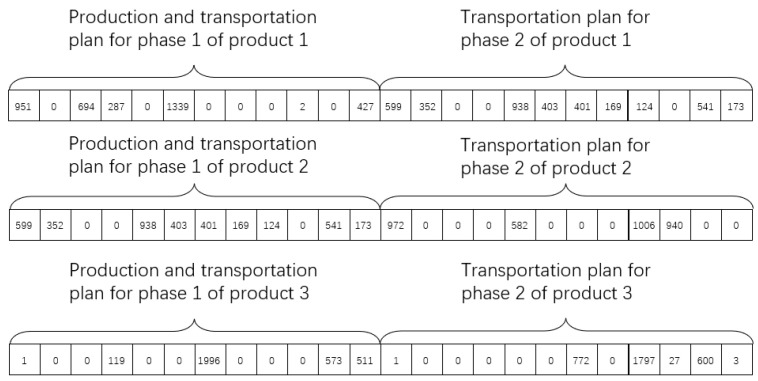
Integrated encoding of decision variables in the whole process.

Method (2): integrated encoding, that is, the distribution strategies of the two stages are encoded into one chromosome. The production solution of the enterprises, the production-distribution solution of the first stage, and the distribution solution of the second stage, are generated simultaneously according to the constraint conditions when the chromosome is randomly initialized. The relationship between the output and input of transit warehouses is maintained by constraints. The relationship is: the quantity of product p shipped from warehouse i to agents in the second stage is equal to the quantity of product p shipped to warehouse i in the first stage. The MVC collaboration network model is built with the experimental parameters in Section 5.3. The possible values of each group of decision variables (MQ, $D_{1}$, and $D_{2}$) are represented by one-dimensional decimal chromosomes as shown in Figure 5.

According to the preliminary analysis of the encoding method, since the staged encoding and solving method minimizes the total cost by stages, there are fewer decision variables and a smaller solution space ranges in each stage, so the optimization effect of the total cost is not as good as the integrated encoding method in theory. However, because of fewer decision variables and more constraints, the solution speed of the staged encoding is faster. In Section 5, a comparative experiment and analysis of these two encoding methods are conducted.

### 4.3. Fitness Function

The fitness function is the standard used to distinguish the advantages and disadvantages of individuals. Because the research is about the optimization effect, the objective function is the total cost of the MVC collaboration network, that is, the sum of the total production cost and the total distribution cost. The calculation formula is shown in Equation (15).

(15) $$fit=F_{1}\left(Q\right)+F_{2}\left(Q,L\right)$$

### 4.4. Selection Operator

The selection operator simulates the survival of the fittest in the natural biological evolution process, selecting individuals with high fitness and eliminating individuals with low fitness. The candidate selection operators are the widely used operators: the enhanced elite tournament selection operator and the roulette selection operator. 

The roulette operator can make it possible for all individuals to be selected. While valuing the elite, it also leaves little chance for non-elite to survive, therefore the diversity will be better than the elite championship. However, at the same time, it will lead to slow population convergence. In Section 5, a comparative experiment of these two operators is conducted.

### 4.5. Crossover Operator

**Figure 6:**
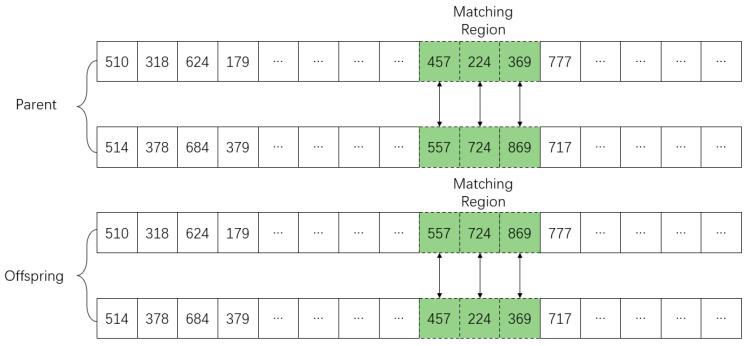
Chromosome crossover operator.

The crossover operator is one of the core elements of the genetic algorithm. By randomly selecting the chromosomes of parents to carry out allelic cross-exchange, the excellent genes of the parents can be passed on to the offspring in a certain probability, to produce excellent offspring in the crossover process. Since the proposed encoding methods have few requirements on crossover operator and the research focus is encoding method and selection operator, a simple two-point crossover-based partial matching crossover operator is adopted, randomly generates matching regions on the alleles of the paternal chromosomes, and exchanges genes of two paternal matching regions according to the crossover probability [46]. The partial matching crossover strategy is shown in Figure 6.

### 4.6. Mutation Operator

**Figure 7:**
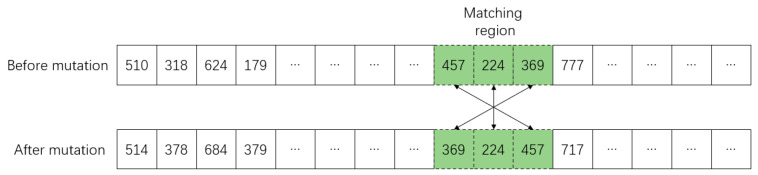
Chromosome mutation operator.

Mutation refers to the mutation of genes on individuals’ chromosomes, thus generating new individuals. Mutation operation is one of the important means of genetic algorithms to produce excellent individuals. The reversal mutation operator [47] is used to randomly generate two reversal points on chromosome individuals, and the gene fragments between the two points are arranged in reverse order to generate new individuals, as shown in Figure 7.

According to the characteristics of production-distribution decision space, a genetic algorithm is used to solve the MVC collaboration network optimization problem. Moreover, according to the specific scenario of production-distribution, two chromosome encoding methods are proposed: staged encoding and integrated encoding. The selection operator, crossover operator, mutation operator are used to solve the problem, and specific methods of chromosome encoding are introduced in this chapter.

## 5. Experiment and Analysis

### 5.1. Experiment Environment

The experiment environment and device configuration are as follows: operating system: Windows 10; processor: Intel Core (TM) i5-7300HQ CPU @ 2.50 GHz; language: Python 3; integrated development environment: JetBrains PyCharm Community Edition.

### 5.2. Data of Experiment

Based on the household appliance industry and combined with the production, distribution, and sales data of a dynamic enterprise alliance, a MVC collaboration network of multiple production ends, multiple agents, and multiple products is constructed. There are 3 enterprises, 4 transit warehouses, and 3 agents in the network, and the simulation experiment is carried out for 3 kinds of products. Specific simulation data are shown in Table 2 of enterprises; Table 3 of transit warehouses; and Table 4 of agents.

### 5.3. Experiment Parameters

#### 5.3.1. Experiment Parameters for Encoding Methods

To ensure the objectivity of the comparison experiment, the algorithm, selection, crossover, and mutation operators used in the experiment are the same except for the encoding method. The algorithm parameters are shown in Table 5.

#### 5.3.2. Experiment Parameters of Algorithms

The compared algorithms are SGA with the roulette operator and SEGA with the elite tournament operator. To ensure the objectivity of the comparison experiment, the two algorithms adopted unified parameters. The algorithm parameters are shown in Table 6.

### 5.4. Analysis of Experiment Results

#### 5.4.1. Analysis of the Results Obtained by Different Encoding Methods

For the staged encoding and integrated encoding, the SEGA algorithm with the same experiment parameters is used to solve the same problem 10 times. The best fitness and solution time of the last generation in ten experiments with different methods were recorded respectively for comparison. The results of different encoding methods are shown in Table 7.

1. Comparative analysis of experiment results

**Figure 8:**
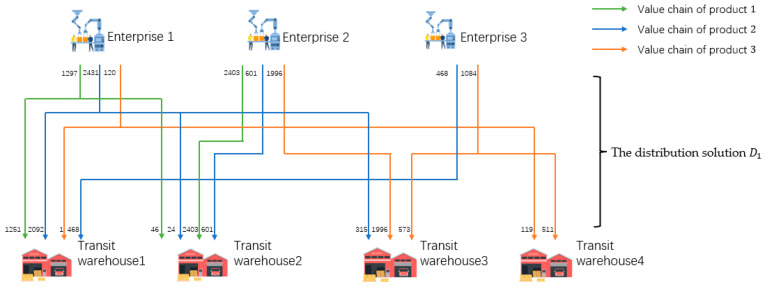
The first stage of production-distribution.

**Figure 9:**
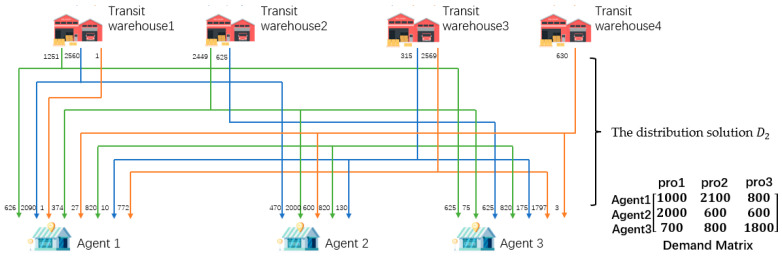
The second stage of production-distribution.

As can be seen from the above results, the optimal fitness of the staged encoding is lower than that of the integrated encoding, because the overall process is divided by the staged encoding into two staged planning problems as shown in Figure 8 and Figure 9.

In the scenario of Section 3.3, on the premise that the constraint conditions of Section 3.6 are satisfied, and the experimental data of Section 5.2 are taken as an example, the distribution strategy with the minimum distribution cost in the first stage of the product is set as shown in (16). In (16), the left matrix is the distribution strategy of product 1, the middle matrix is the distribution strategy of product 2, and the right matrix is the distribution strategy of product 3. $d_{2}^{3}$ means d pieces of products shipped from enterprise 2 to transit warehouse 3.

(16) $$\left[\left.\begin{array}{cccc} 1251 & 46 & 0 & 0\\ 0 & 2403 & d_{2}^{3} & 0\\ 0 & 0 & 0 & 0 \end{array}\right|\begin{array}{cccc} 2092 & 24 & 315 & 0\\ 0 & 601 & 0 & 0\\ 468 & 0 & 0 & 0 \end{array}\left|\begin{array}{cccc} 1 & 0 & 0 & 119\\ 0 & 0 & 1996 & 0\\ 0 & 0 & 573 & 511 \end{array}\right.\right]$$

The distribution solution of the second stage is shown in (17). Matrices (16) and (17) are staged solutions with the lowest cost of each stage as the optimization objective function. The integrated method takes the total cost of the entire MVC collaboration network as the optimization objective function. Therefore, there is a solution D in this method in theory: the production and distribution cost of the first stage of D is higher than that of (16), but the total cost of D is lower than the summary cost of (16) and (17).

(17) $$\left[\begin{array}{c} \left.\begin{array}{cc} \begin{array}{cc} 626 & 0\\ 374 & 2000\\ 0 & 0\\ 0 & 0 \end{array} & \begin{array}{c} 625\\ 75\\ 0\\ 0 \end{array} \end{array}\right| \end{array}\begin{array}{ccc} 2090 & 470 & 0\\ 0 & 0 & 625\\ 10 & 130 & 175\\ 0 & 0 & 0 \end{array}\left|\begin{array}{ccc} 1 & 0 & 0\\ 0 & 0 & 0\\ 772 & 0 & 1797\\ 27 & 600 & 3 \end{array}\right.\right]$$

2. Comparative analysis of the evolution process

**Figure 10:**
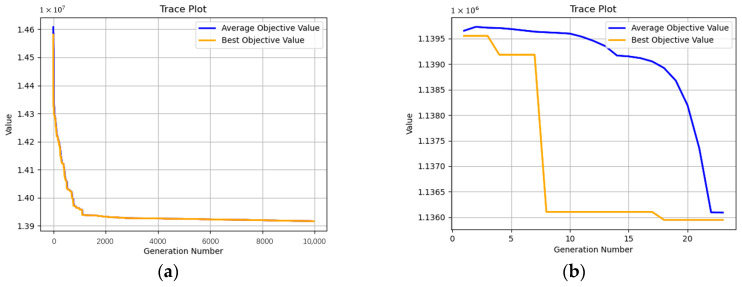
The evolution processes of staged methods: (**a**) the first stage of staged encoding; (**b**) the second stage of staged encoding.

In terms of the speed of the solution, because the optimization is carried out in stages, each stage has more constraints, fewer decision variables, and a smaller solution space, so the speed of the solution is faster. This feature is more prominent in the comparison of the evolution process in the first and second stages (Figure 10).

**Figure 11:**
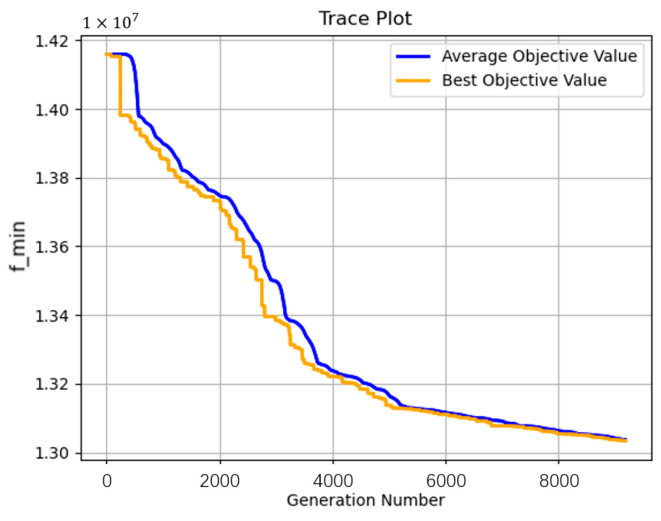
The evolution process of integrated encoding.

Based on the experiment results and the images of the evolution processes (Figure 10 and Figure 11), it can be concluded that staged encoding is faster than integrated encoding because of more constraints, smaller optimization space, and fewer decision variables, so it is more suitable for the MVC network with multiple products where the time cost is longer. Besides, it is easy to assign different constraints to the MVC network in different stages according to the requirements of nodes, so the staged encoding is suitable for complex scenarios that have many requirements on the distribution quantity and time at each stage. However, in terms of the optimization, the staged encoding is inferior to the integrated encoding, because the integrated encoding is used to optimize the entire production and distribution process, which has more decision variables and larger optimization space. Although integrated encoding needs longer solving time, the cost of the optimization result is 7% lower on average than that of the staged encoding. So, integrated encoding is suitable for scenarios where cost reduction is given priority and there are no strict requirements for each stage of the value chain.

#### 5.4.2. Comparative Analysis of Solving Algorithms

1. Comparative analysis of SGA and SEGA

**Figure 12:**
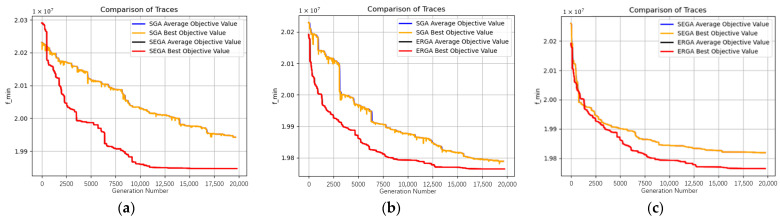
The evolution processes of algorithms: (**a**) processes of SEGA and SGA; (**b**) processes of ERGA and SGA; (**c**) processes of SEGA and ERGA.

With the above algorithm parameters and the integrated encoding method adopted, the same problem is solved 10 times by both the SEGA algorithm, which adopts the elitist tournament operator, and by the SGA algorithm, which adopts the roulette operator. The worst fitness, optimal fitness, algorithm time cost, and fitness standard deviation of the last generation in ten experiments of different algorithms were recorded respectively for comparison. The results of different algorithms are shown in Table 8 and Figure 12.

Figure 12a shows the images of the population evolution process of SGA and SEGA, with the horizontal coordinate as the number of iterations, and the vertical coordinate as the minimum value of population fitness value, i.e., the minimum value of total cost. Since the minimum value of each generation is close to the mean value, the optimal value curve covers the mean curve. From the solution results, it can be seen that the cost of SEGA is lower than that of SGA in nine out of ten experiments, and the standard deviation of SEGA is lower than that of SGA each time, indicating that the stability of SEGA is better and the diversity of SGA is better; from the comparison of the solution process images, it can be seen that SGA has not reached the convergence state at 20,000 generations, and SEGA has reached the local convergence state at the 12,000th generation. It can be concluded that SEGA has a better convergence speed compared with SGA but is more likely to fall into local convergence. Since SEGA has an elite retention after merging the pre-variant population with the post-variant population, the computation time of each generation of SEGA is longer, but SEGA converges faster. The reason for the transient fluctuations in the optimal target value curve of SGA is that the roulette selection operator unnecessarily selects the optimal individual.

2. Comparative analysis of ERGA and SGA

Since the tournament operator combined with elite retention performs better than the roulette operator, this section will further study the influence of elite retention strategy on the roulette operator based on the scenario above and compare the performance of ERGA and SGA, which is enhanced by elite retention. The experimental results of ERGA and SGA are shown in Table 8 and Figure 12b.

According to the evolution process images in Figure 12 and the experimental results in Table 8, it can be concluded that compared with the original SGA, ERGA reserves more elite individuals, and has a faster convergence rate. However, at the same time, because dominant individuals will be copied and quickly spread to the population, the diversity of SEGA will be reduced, and it is easier to fall into local convergence. Also, because of the extra time needed to join the parent population and sort it, ERGA would be more time-consuming than the original SGA.

3. Comparative analysis of ERGA and SEGA

Finally, ERGA and SEGA, both of which combine the elite retention strategy, are compared and analyzed. The experimental results of ERGA and SEGA are shown in Table 8 and Figure 12c. According to the evolutionary process images in Figure 12c and the experimental results in Table 8, after being enhanced by the elite retention strategy the performance of the SGA algorithm has been significantly improved. The quality of the optimization solution obtained with the same evolutionary generation has changed from a big gap with SEGA to a small gap or even better than SEGA. It can be concluded that the elite retention strategy improves the convergence speed of ERGA and the diversity of the roulette operator is not significantly reduced due to the low percentage of elite retention. Therefore, ERGA explores the solution space more fully and optimizes better. However, ERGA is required to score and rank the fitness of all individuals, while SEGA only needs to rank the individuals selected for tournament play, so ERGA is larger than SEGA in terms of time cost. 

#### 5.4.3. Analysis of the Solution

As can be seen from the above comparative analysis, the elite retention strategy can improve the convergence of the algorithm by retaining more elite individuals, but it also reduces the diversity of the algorithm and makes it easier to fall into local convergence. Compared with SEGA, which also combines an elite retention strategy, ERGA performs better in terms of global convergence with little time difference. 

**Figure 13:**
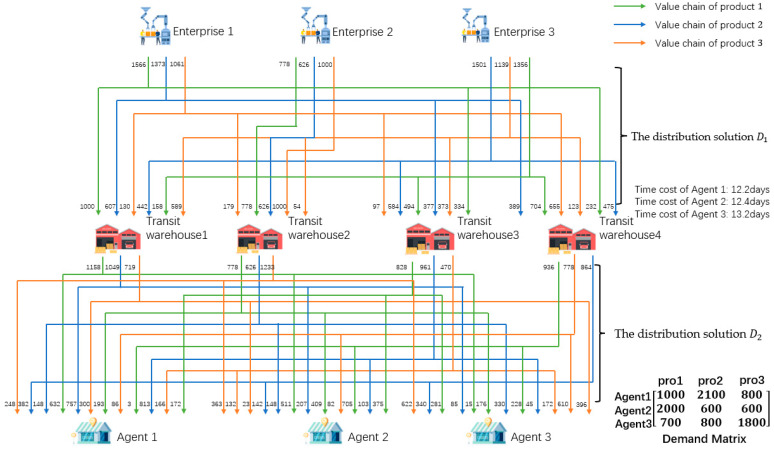
Optimization results of production and distribution solution.

In the scenario of Section 3.3, the primary goal of the MVC collaboration optimization network is to reduce the total cost of production and distribution. Therefore, according to the above conclusion, the ERGA algorithm combined with the integrated encoding method should be selected. The production solution is shown in Figure 13.

Specifically, the first stage of the solution is shown in Table 9, and the second stage of the solution is shown in Table 10. Taking enterprise 1 as an example; enterprise 1 produced 1566 pieces of product 1, then delivered 1000 pieces to transit warehouse 1, 334 pieces to warehouse 3, and 232 pieces to warehouse 4. Enterprise 1 produced 1373 pieces of product 2, then delivered 607 pieces to transit warehouse 1, 377 pieces to warehouse 3, and 389 pieces to warehouse 4. Enterprise 1 produced 1061 pieces of product 3, then delivered 130 pieces to transit warehouse 1, 179 pieces to warehouse 2, 97 pieces to warehouse 3, and 655 pieces to warehouse 4.

From Figure 13, Table 9 and Table 10, it can be concluded that the ERGA algorithm, which combines the integrated coding approach, yields a multi-product production-distribution solution that meets the requirements of the manufacturers, transit warehouses, and agents in each value chain without node conflicts.

In this chapter, based on the background of the household appliance industry and combined with the production, distribution, and sales data of a dynamic enterprise alliance, an MVC collaboration network of multiple products, multiple production ends, and multiple agents is constructed. Due to the multi-stage characteristic of the production-distribution scenario, two chromosome coding methods are proposed in this paper: staged coding and integrated coding. The comparative experimental results and evolutionary process of the coding methods show that staged coding is more suitable for complex scenarios with requirements on the number of distributions and distribution time at each stage. Integrated coding is more suitable for scenarios where cost reduction is a priority and there are no strict requirements for each stage of the value chain.

Since the tournament operator combined with elite retention performs better than the roulette operator, the influence of elite retention strategy on the algorithm has been further studied. A comparative experiment analysis for SGA and SEGA genetic algorithms is conducted and it shows that the elite retention strategy can improve the convergence of the algorithm by retaining more elite individuals, but it also reduces the diversity of the algorithm and makes it more likely to fall into local convergence. Based on this, a roulette algorithm ERGA combined with the elite retention strategy is proposed. The comparative experiment results and the analysis of the population evolution process show that ERGA has the best combined effect in terms of time cost and optimization compared with SGA and SEGA.

## 6. Conclusions

In the enterprise dynamic alliance, an analysis of the essential characteristics of the MVC collaboration network has been conducted. An MVC collaboration network optimization model aiming at the optimization demand of the MVC collaboration network in production-distribution is proposed. It takes production-distribution solutions as the decision space, the total cost as the optimization objective, and the time limit and capacity as constraints. For the high-dimensional characteristics of the decision space in the multi-task, multi-production end and multi-distribution end scenario, a genetic algorithm is used to solve the MVC collaboration network optimization model. The collaboration constraints of nodes in the chains are mapped as constraints among genes of chromosomes, and the problem of difficult collaboration or even conflicts among nodes in MVC collaboration networks is solved by adjusting the constraints among genes. In view of the multi-level characteristics of the production-distribution scenario, two chromosome coding methods are proposed in this paper: staged coding and integrated coding. The comparative experiments show that the staged coding is more suitable for complex scenarios with high requirements on distribution volume and time at stages; the integrated coding is more suitable for the optimization scenarios pursuing lower costs in the whole production-distribution process. Finally, this paper proposes a roulette algorithm ERGA, which was combined with the elite retention strategy. The comparative experimental results and the analysis of the population evolution process show that ERGA outperforms SGA and SEGA in terms of time cost and optimization, and that ERGA is more generalized. By reasonably combining coding methods and selection operators, the MVC collaboration network optimization model can be made more adaptable to different production-distribution environments and help the dynamic alliance of enterprises to solve the optimal solution under different production-distribution high-dimensional environments.

In future research, the influence of the fullness of the TEU (transmission extension unit) on the transportation cost could be introduced into the optimization model, and the matching modeling analysis of the fullness and the shipping volume could be conducted so that the scenario is more in line with the actual transportation cost, since this paper does not consider the influence of the fullness of the TEU on the transportation cost. Secondly, based on the research scenario of product production-distribution in this paper, which involves product manufacturing, warehousing, logistics, etc., it could be further extended to carry out research on the scenario of the MVC of the whole product process, including raw material supply, product manufacturing, warehousing, logistics, marketing, and sales, etc., involving the whole process of the MVC collaboration planning of suppliers, manufacturers, distributors, and customers.

## Figures and Tables

**Table 1:** Symbol table.

Symbol	Description	Symbol	Description
VC	Collection of value chains	$MaxQ_{tw_{i}}$	The maximum storage quantity of transit warehouse $tw_{i}$
$VC_{i}$	The i-th value chain	$T_{a_{i}}$	The maximum lead time of agent $a_{i}$
$n_{i}^{j}$	The j-th value node on the i-th value chain	TC	The total cost of the MVC collaboration network
P	Collection of products	TPC	The total production cost of the MVC collaboration network
$P_{tol}$	Total number of products	TFC	Total fixed cost of the MVC collaboration network
$p_{i}$	The products of the i-th category, $p_{i}\in P$	TTC	Total transportation cost of the MVC collaboration network
E	Collection of enterprises	$C_{e_{i}}^{p_{i}}$	Daily capacity of enterprise $e_{i}$ to produce product $p_{i}$
$E_{tol}$	Total number of enterprises	$T_{e_{i}}^{p_{i}}$	Time for enterprise $e_{i}\;$ to complete the order of product $p_{i}$
e	The e-th enterprise, e∈E	$T_{tw_{i}}^{e_{i}}$	The time it takes for products to be distributed from enterprise $e_{i}$ to transit warehouse $tw_{i}$
TW	Collection of transit warehouses	$T_{a_{i}}^{tw_{i}}$	The time it takes for products to be distributed from transit warehouse $tw_{i}$ to agent $a_{i}$
$TW_{tol}$	Total number of transit warehouses	$F_{1}$	Mapping function between variable cost and product quantity
$tw_{i}$	The i-th transit warehouse, tw∈TW	$F_{2}$	Mapping function of distribution cost, product quantity, and path length
A	Collection of agents	L	Matrix of path length
$A_{tol}$	Total number of agents	$l_{i}^{j}$	The path length from node i to node j
$a_{i}$	The i-th agent, $a_{i}\in A$	fit	Fitness function
$D_{1}^{p_{i}}$	Distribution solution in the first stage of product $p_{i}$	$Q_{e_{i}}^{p_{i}}$	Number of product $p_{i}$ produced by enterprise e
$d_{e_{i}}^{tw_{j}}$	Total number of the products distributed from enterprise $e_{i}$ to transit warehouse $tw_{j}$	$Q_{tw_{i}}^{p_{i}}$	Number of product $p_{i}$ stored in transit warehouse $tw_{i}$
$D_{2}^{p_{i}}$	Distribution solution in the second stage of product $p_{i}$	$Q_{a_{i}}^{p_{i}}$	Quantity of product $p_{i}$ purchased by agent $a_{i}$
$d_{tw_{i}}^{a_{j}}$	Total number of the products distributed from transit warehouse $tw_{j}$ to agent $a_{j}$		

**Table 2:** Information of the enterprises.

Enterprise No.	MC (¥ Per Piece)	FC (Million ¥)	$C_{e}^{p}$, (Daily Production)		
			Product 1	Product 2	Product 3
1	1550	0.5	1000	900	1100
2	1650	0.7	1100	700	1000
3	1600	0.6	1200	1000	900

**Table 3:** Information of the transit warehouses.

Warehouse No.	$MaxQ_{t}$ (Pieces)	$D_{t}^{e}$ (km)		
		Enterprise 1	Enterprise 2	Enterprise 3
1	4000	100	2500	2300
2	3500	2200	800	2000
3	3000	2000	1400	1800
4	3500	1500	1600	1500

**Table 4:** Information of the agents.

Agent No.	$T_{c}$ Per Day	$Q_{c}^{p}$, (Pieces)			$D_{c}^{t}$, (km)			
		Product 1	Product 2	Product 3	Ware- House 1	Ware- House 2	Ware- House 3	Ware- House 4
1	15	1000	2100	800	500	2300	2400	850
2	15	2000	600	600	3000	900	1800	1800
3	15	700	800	1800	3600	1700	700	2870

**Table 5:** Experiment parameters for the encoding methods.

Encoding Method	Population Size	Algorithm	Evolutionary Generations	Crossover Probability	Mutation Probability
Staged encoding	5000	SEGA	20,000	90%	20%
Integrated encoding	5000	SEGA	20,000	90%	20%

**Table 6:** Algorithm parameters.

Algorithm	Population Size	Evolutionary Generations	Crossover Probability	Mutation Probability
SGA	5000	20,000	90%	20%
SEGA	5000	20,000	90%	20%

**Table 7:** Experiment data of the encoding methods.

$\mathrm{Best}\;\mathrm{Fitness},\;f_{min}$ (¥)			Time Cost (s)	
Staged Encoding	Integrated Encoding	Gap of Best Fitness	Staged Encoding	Integrated Encoding
21,034,179	19,249,951	8.48%	609.305	1033.66
21,072,627	19,969,750	5.23%	610.713	1035.62
21,083,618	20,041,390	4.94%	617.838	1051.23
21,155,280	19,345,940	8.55%	588.161	1088.91
21,987,913	19,460,975	11.49%	592.410	1052.95
21,037,718	19,950,505	5.17%	608.124	1033.63
21,020,710	19,109,735	9.09%	583.551	1039.44
20,958,410	19,771,720	5.66%	572.628	1048.78
21,063,779	20,025,090	4.93%	566.634	1051.49
21,143,800	19,870,990	6.02%	579.786	1037.93

**Table 8:** Experiment results of SGA, SEGA, and ERGA.

Best Fitness, f_min (¥)			Worst Fitness, f_max (¥)			Time Cost (s)			Standard Deviation of Fitness, f_std		
SGA	SEGA	ERGA	SGA	SEGA	ERGA	SGA	SEGA	ERGA	SGA	SEGA	ERGA
20,030,187	19,849,478	19,651,383	21,285,757	20,086,978	19,888,883	935.75	1065.37	1228.26	134,024.43	23,494.22	21,038.63
19,907,146	19,809,604	19,703,741	21,233,376	20,147,104	19,941,241	928.36	1059.46	1221.01	139,240.78	25,064.57	24,736.33
19,954,510	19,803,118	19,912,110	21,387,070	19,974,908	20,151,510	958.66	1093.96	1244.24	136,504.67	18,943.18	27,164.67
19,992,657	19,892,734	19,760,671	21,328,307	20,130,234	19,998,171	913.71	1098.42	1297.24	137,321.44	24,284.71	24,668.79
19,913,814	19,909,604	19,657,517	21,422,150	20,147,104	19,883,767	899.18	1059.46	1296.35	147,413.45	25,064.57	17,993.85
19,971,522	19,849,478	19,756,053	22,569,942	20,086,978	19,981,053	908.85	1065.37	1287.20	141,977.68	23,494.22	22,493.11
19,992,806	19,882,079	19,868,010	21,844,706	20,119,579	20,093,010	933.69	1044.54	1267.65	150,292.57	23,112.23	19,900.79
19,949,906	19,944,644	19,764,781	21,389,986	20,182,144	20,002,281	875.98	1100.74	1253.51	151,597.91	24,861.79	25,712.40
19,965,013	19,943,682	19,743,073	21,567,413	20,122,282	19,980,573	881.38	1074.60	1270.67	139,943.68	19,186.87	24,335.14
19,954,700	19,955,039	19,887,158	21,304,590	20,177,539	20,124,658	867.82	1073.79	1267.19	141,423.25	20,093.23	20,301.43

**Table 9:** The first part of the solution.

	Product 1				Product 2				Product 3			
	Warehouse 1	Warehouse 2	Warehouse 3	Warehouse 4	Warehouse 1	Warehouse 2	Warehouse 3	Warehouse 4	Warehouse 1	Warehouse 2	Warehouse 3	Warehouse 4
Enterprise 1	1000	0	334	232	607	0	377	389	130	179	97	655
Enterprise 2	0	778	0	0	0	626	0	0	0	1000	0	0
Enterprise 3	158	0	494	704	442	0	584	475	589	54	373	123

**Table 10:** The second part of the solution.

	Product 1			Product 2			Product 3		
	Agent 1	Agent 2	Agent 3	Agent 1	Agent 2	Agent 3	Agent 1	Agent 2	Agent 3
Warehouse 1	632	511	15	757	207	85	300	23	396
Warehouse 2	193	409	176	148	148	330	248	363	622
Warehouse 3	172	375	281	813	103	45	166	132	172
Warehouse 4	3	705	228	382	142	340	86	82	610

## Data Availability

Not applicable.
